# Preparation, Characterization, and Evaluation of β‐Cyclodextrin Inclusion Hydrogel Films Based on Dates Carboxymethyl Cellulose for Hydrophobic Drug Delivery

**DOI:** 10.1002/cbdv.71622

**Published:** 2026-08-31

**Authors:** Loubna Oumamar, El Khamsa Soltani, Kailas Krishnat Mali, Ahlem Karbab, Noureddine Charef, Nancy Sowan, Mohammad S. Mubarak

**Affiliations:** ^1^ Laboratory of Applied Biochemistry Setif‐1 University of Ferhat Abbas 19000 Algeria; ^2^ Department of Pharmaceutics Adarsh College of Pharmacy Vita Maharashtra India; ^3^ Department of Chemistry The University of Jordan Amman Jordan

**Keywords:** anti‐inflammatory activity, carboxymethyl cellulose, hydrogel films

## Abstract

This work investigates the carboxymethylcellulose (CMC)‐dates (Dt) hydrogel films (HFs) for the controlled delivery of hydrophobic drugs, using citric acid (CA) as a nontoxic cross‐linking agent. The model drug Econazole (ECN) was loaded into HFs, and drug release was studied at pH 7.4. The HFs were evaluated for β‐cyclodextrin (β‐CD) content, carboxyl content, swelling ratio, drug loading, drug release, and in vivo anti‐inflammatory activity. These films were characterized using ATR‐FTIR, thermal gravimetric analysis (TGA), scanning electron microscopy coupled with energy‐dispersive x‐ray spectroscopy (SEM/EDX), and atomic force microscopy (AFM) for morphological measurements. Results revealed that the HFs with high carboxyl content exhibit maximum swelling and high drug loading. The incorporation of β‐CD helped to retard the release of ECN from the HFs. Our findings indicate that HFs F3 and F4 with high Dt concentrations reduce the edematous response by 92% ± 5.07% and 89% ± 3.69%, respectively. Altogether, our findings show that CMC‐Dt HFs are suitable for the delivery of non‐soluble weak bases and have potential for wound‐healing applications. The in vivo investigation demonstrated that the HFs exhibit significant anti‐inflammatory characteristics.

## Graphical abstract

We prepared carboxymethylcellulose (CMC)‐date (Dt) hydrogel films using citric acid (CA) as a cross‐linking agent. These CMC‐Dt hydrogel films exhibited high swelling ability and good drug loading with regulated release of econazole (ECN). It also exhibited excellent topical anti‐inflammatory activity.

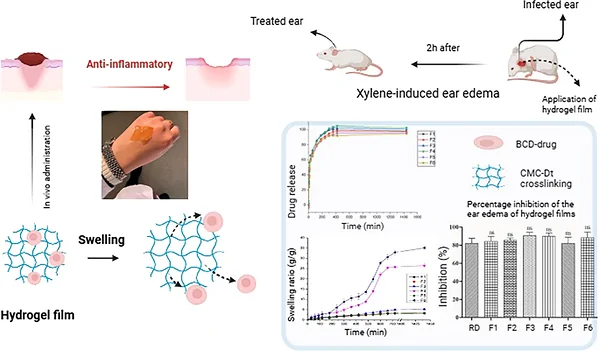

## 1 Introduction

Hydrogels are 3D cross‐linked networks, and their chemical and physical linkages render them insoluble in water, although they can absorb large amounts of water [1, 2]. Their high‐water content and soft structure confer excellent biocompatibility, making them attractive drug‐delivery systems with broad biomedical and pharmaceutical applications [3, 4]. Hydrogels can be classified as natural or synthetic [5]. Natural hydrogels possess advantageous properties, such as biocompatibility and biodegradability [6], and are more economical than synthetic hydrogels. Natural polymers used to make hydrogels include cellulose [7], starch [8], and chitosan [9].

Cross‐linking can be achieved through physical or chemical methods, with chemical cross‐linking generally providing improved mechanical stability compared to physically cross‐linked systems [10]. Among natural polymers, polysaccharides, especially cellulose, have attracted considerable attention because of their abundance and versatility, making them promising materials for hydrogel‐based drug delivery systems [11, 12, 13].

Among cellulose derivatives, carboxymethylcellulose (CMC) is inexpensive, nontoxic, biodegradable, water‐soluble, and biocompatible [14]. Numerous studies have addressed the use of cellulose‐based hydrogels for controlled drug delivery [15, 16, 17, 18]. Despite the ability of cyclodextrin inclusion complexes to enhance hydrophobic drug loading in hydrogel, their propensity to diffuse out of the hydrogel network can hinder controlled and sustained release performance [19, 20]. To overcome this limitations, several researchers prepared cellulose‐based hydrogels using β‐cyclodextrin (β‐CD) grafting [21, 22, 23, 24]. Additionally, the reliance on toxic cross‐linking agents in conventional cellulose‐based hydrogels significantly limits their biomedical potential [25]. Consequently, the development of biocompatible hydrogel systems that ensure efficient drug encapsulation and stable, prolonged release poses significant challenges for biomedical applications. To address this issue, the present study employs citric acid (CA) as a nontoxic cross‐linking agent. CA is a low‐cost multifunctional monomer whose hydroxyl and carboxyl groups enable the grafting of β‐cyclodextrin (β‐CD) onto cellulose and the formation of ester cross‐links, improving network stability and drug complexation [26, 27, 28, 29]. These interactions enhance the loading of poorly soluble basic drugs [19, 30]. In contrast, CMC alone forms weakly cross‐linked hydrogels. To overcome this, an additional polysaccharide is typically required to strengthen the matrix [31, 32, 33].

We hypothesized that incorporating β‑CD into CMC‑Dt hydrogel films (HFs) using CA would enhance drug encapsulation efficiency, improve the release kinetics of hydrophobic drugs (ECN), and provide measurable anti‑inflammatory efficacy. The HFs were designated as F1, F2, F3, F4, F5, and F6. The presence or absence of β‐CD and the concentration of Dt were varied in the HFs to assess their effects on the hydrogel properties. Econazole (ECN) is widely used as a topical antifungal, incorporated into hydrogels as a basis for wound and topical drug delivery systems. For these reasons, ECN was selected for testing the efficiency of the CMC/Dt HFs in trapping lipophilic compounds and releasing them.

Several studies have explored β‐CD polysaccharide hydrogels to enhance drug loading and to control drug release [34, 35, 36, 37]. In this study, we combined CMC and DT. The novelty of this study lies in the synergistic integration of date‐derived polysaccharides (Dt) with CMC and β‐CD (Table 1). This combination, facilitated by CA cross‐linking, uniquely exploits the hydroxyl‐rich composition of Dt, which is expected to enhance cross‐linking density and mechanical integrity, and potentially improve the swelling, drug encapsulation, and sustained‐release profiles beyond those of conventional β‐CD–CMC systems typically prepared without Dt or using different cross‐linking strategies. Dt, which is rich in starch and cellulose [31], can uniquely contribute abundant hydroxyl groups to CA‐mediated esterification, potentially resulting in a higher cross‐linking density than that of conventional β‐CD–CMC hydrogels. The sugar matrix enhances swelling and drug encapsulation effectiveness, while the fiber content supports mechanical integrity. Thus, Dt serves as a functional polysaccharide component rather than a nutritional additive. Dt was employed as a commercially supplied powder (Nakheel Alya Dates Factory) without further extraction or purification steps. Although this strategy highlights the practical applicability of the system, subsequent studies will aim to isolate and characterize specific polysaccharide fractions to ensure improved reproducibility and suitability for pharmaceutical applications.

**TABLE 1:** Comparison with prior work.

Studies	Polymer System	Cross‐linker	β‑CD incorporation	Unique Features
Ghorpade et al., 2017	CMC + β‑CD	CA	Grafting	Controlled release for hydrophobic drugs
Jeong et al., 2018	CMC + cmβ‑CD	/	Grafting	Mechanical strength and effective drug delivery
Naeem et al., 2023	HPMC + βCD	/	Grafting	Controlled drug delivery
Present work	CMC + β‑CD + Dt	CA	Grafting + Dt polysaccharides	Enhanced cross‐link density, drug loading, and anti‐inflammatory efficacy

## 2 Materials and Methods

### 2.1 Materials

The chemicals and reagents used in this study were obtained from commercial sources and used as received. Econazole (ECN, C_18_H_15_C_l3_N_2_O, molecular weight: 381.684 g/mol, ≥ 98%), sodium carboxymethylcellulose (CMC, degree of substitution: 0.7, average molecular weight: ∼250000, ≥ 99%), citric acid (CA, anhydrous, ≥ 99.5%), and β‐cyclodextrin (β‐CD, ≥ 98%), were purchased from Sigma‐Aldrich (St. Louis, MO, USA), Dates (Dt, a commercial product by Nakheel Alya Dates Factory). All additional chemicals were categorized as analytical. All reagents were used as provided.

### 2.2 Preparation of β‐CD‐CMC HFs

HFs were prepared according to literature procedures with some modifications [10, 38, 39]. At room temperature, CMC (2%) aqueous solutions were stirred with β‐CD, Dt, and CA as a cross‐linking agent, as detailed in Table 2. The solutions were left overnight to eliminate air bubbles. The transparent solutions were poured into uniformly sized Petri dishes (9 cm in diameter) and allowed to dry for 24 h at 50°C in a hot air oven. The dry films were cured at 140°C for five minutes. Cross‐linking can be achieved at the appropriate curing temperature and duration [40, 41]. After the HFs were dried, they were repeatedly cleaned with distilled water until the pH of the water was almost neutral, and then with isopropyl alcohol for 1 h to remove unreacted substances [10, 42]. The HFs were then placed in a desiccator after drying for 24 h at 50°C in a hot‐air oven. The concentrations of CMC and Dt were varied to investigate their impact on the film characteristics, as shown in Table 2.

**TABLE 2:** CMC‐Dt HF composition.

Parameters	HF code					
	F1	F2	F3	F4	F5	F6
CMC: Dt^a^	1:1	1:1	1:2	1:2	2:1	2:1
β‐CD (%)	0.4	/	/	0.4	/	0.4
CA (%)	20	20	20	20	20	20
Temperature (°C)	140	140	140	140	140	140
Curing time (min)	5	5	5	5	5	5

Abbreviations: Dt, Dates; CMC, carboxymethyl cellulose (sodium salt); β‐CD, β‐cyclodextrin, CA, Citric acid.

^a^concentration of total polymers equal to 2% *w*/*v*; *w*/*v*.

### 2.3 Characterization of the HF

Several analytical techniques were used to characterize HF. The IR spectra of CMC, β‐CD, CA, Dt, ECN, and HF were acquired using an attenuated total reflectance Fourier‐transform infrared (ATR‐FTIR) spectrophotometer (Shimadzu, IR Affinity, Japan). The ATR compartment received the samples for examination, and spectra were obtained at a resolution of 2 cm^−1^, averaging five scans over the 400–4000 cm^−1^ range. The surface morphology of HF was studied using scanning electron microscopy (SEM), and the elemental composition was analyzed by energy‐dispersive x‐ray spectroscopy (EDX) integrated with the SEM system (FEI QUANTA 250, UK). The samples were placed on double‐sided tape, sputter‐coated with platinum, and photomicrographs of the HFs were obtained at an acceleration voltage of 5 kV. Morphological measurements (AFM) of the HFs were performed using an Asylum Research MFP‐3D microscope. Small film portions were attached to magnetic sample holders placed on the scanner tube and adhered to metal disks. Images were captured using cantilevers operating in intermittent‐contact mode at the Molecular Engineering and Nanostructure Chemistry Laboratory (LCIMN) at Ferhat Abbas University—Sétif 1. Image processing was performed using RMS and standard statistical software. Thermogravimetric analysis (TGA) of the HF was performed using SDT Q600 (TA Instruments). The samples were heated from 30°C to 600°C under a nitrogen atmosphere (flow rate: 100 mL/min) at a heating rate of 10°C/min.

### 2.4 Determination of β‐CD Content

The amount of β‐CD bound to the CMC network in the hydrogels was estimated using a phenolphthalein assay [43]. To construct the calibration curve, 0.05 M trisaminomethane hydrochloride (Tris HCl) buffer (pH 7) was used to prepare standard solutions of β‐CD (0.1–0.5 mM/mL). Briefly, 4 mL of the working phenolphthalein solution was combined with 1 mL of the reference solutions. Sample solutions comprising HF (0.1 g) were prepared by mixing 1 mL of Tris‐HCl buffer with 4 mL of working phenolphthalein solution in test tubes. The test tubes were vortexed and left in the dark for 2 h. The absorbances of the standard and sample solutions were measured at 550 nm using a UV–vis spectrophotometer (UV‐7315 JENWAY). The following formula was used to determine the percentage decrease in absorbance of the solutions:

(1) $$\%\;\;\mathrm{Decrease}\;\mathrm{in}\;\mathrm{absorbance}=\frac{Abs_{\mathrm{control}}-Abs_{\mathrm{test}}}{Abs_{\mathrm{control}}}$$

Standard solutions were prepared by mixing 1 mL Tris‐HCl buffer with 4 mL of working phenolphthalein solution, whereas sample solutions were prepared from samples containing ungrafted HFs. The amount of β‐CD was calculated from a calibration curve relating the percentage decrease in absorbance to β‐CD concentration for standard solutions. The procedure was performed in triplicate. The following formula was used to determine the amount of β‐CD in the CMC matrix:

(2) $$\mathrm{Active}\;\beta -\mathrm{CD}\;\mathrm{content}\;\left(\mathrm{mg}/g\;\mathrm{of}\;\mathrm{hydrogel}\right)=\frac{C_{\beta \mathrm{CD}}}{W_{\mathrm{HG}}}\;\;\;\times \;M(\beta -\mathrm{CD})$$

with *M*_β‐CD_ as the molecular weight of β‐CD (g/mol), *W*_HG_ as the weight of the HF (mg), and *C*_βCD_ as the concentration of active β‐CD (mM).

### 2.5 Determination of Carboxyl Content

The carboxyl content of HF was determined by acid–base titration [8, 44]. In this method, a known quantity of HF was dissolved in 0.1N NaOH and stirred for 2 h using a magnetic stirrer. Sodium carboxylate (citrate) is formed when sodium hydroxide breaks down the ester bonds and combines with the free carboxyl groups. Then, 0.1N HCl was used to titrate the excess 0.1N NaOH using phenolphthalein as an indicator. The following formula was used to calculate the total carboxyl content (TCC), expressed as milliequivalents per 100 g of HFs:

(3) $$\mathrm{TCC}\;\left(\mathrm{mEq}/100\;g\;\mathrm{hydrogel}\right)=\frac{\left(V_{b}\;-V_{a}\right)\times \;N\;\times \;100}{w}$$

where *W* is the sample weight (g), *V*_b_ is the volume of HCl without the sample, *V*_a_ is the volume of HCl with the sample, and *N* is the normality of HCl (eq/L) [13].

### 2.6 Determination of Swelling Ratio

The swelling ratio (SR) of the HFs was calculated using published procedures [9, 45, 46]. Briefly, 0.2 g of HF was immersed in 10 mL Tris‐HCl buffer (pH 7.4) at ambient temperature (25°C) [10]. Swelling was evaluated at pH 7.4 and 37°C to simulate physiological conditions and assess the hydrogel's behavior under biologically relevant conditions. These parameters were selected to provide a realistic estimation of the in vivo swelling performance. Further studies across a range of pH environments will be conducted in the future to better understand environmental responsiveness. After 15 min, the enlarged samples were removed from the buffer, and any excess buffer was wiped with tissue paper. The following formula was used to determine the weight of the swelled hydrogel (*W*_s_):

(4) $$\mathrm{Swelling}\;\mathrm{ratio}\;\left(g/g\right)=\frac{W_{s}\;-\;W_{d}}{W_{d}\;}\;$$

*W*_d_ and *W*_s_ are the weights of the dry and swollen hydrogels, respectively [41].

### 2.7 Anti‐Inflammatory In Vivo Investigation

#### 2.7.1 Animals

Adult female mice (25–30 g) purchased from the Pasteur Institute of Algeria were used throughout this study. These animals were housed in cages at a constant room temperature (25°C ± 1°C) for 7 days before the experiments under standard conditions of a light/dark cycle of 12:12. They were given free access to a standard diet and water, and kept under standard conditions mentioned in the Animals By‐Laws No. 425–2008, dealing with the guidelines for the treatment of animals. The experimental assay was approved by the Committee of the ‘Association Algerienne des Sciences en Experimentation Animale’ (http://aasea.asso.dz/ articles/) under law no. 88‐08/1988, associated with veterinary medical activities and animal health protection (N° JORA: 004/1988).

#### 2.7.2 Xylene‐Induced Ear Edema in Mice

The anti‐inflammatory activity of the hydrogel was investigated using the xylene‐induced ear edema method [47, 48]. Mice were randomly divided into eight groups of six animals each (*n* = 6). Mice were topically treated with 30 µL of xylene to induce inflammation. All groups were allowed free access to drinking water before the experiment. Group I, used as a positive control, received topical Voltaren Gel (reference drug, RD), whereas Group II, used as a negative control, received no therapy to determine the baseline inflammatory response. Groups III–VIII (treated groups) received hydrogel samples (drug‐free formulations) from F1 to F6 on the right ear. The anti‐inflammatory evaluation was performed using drug‐free hydrogels as a preliminary assessment to demonstrate the hydrogel matrix's biocompatibility and its intrinsic effect (specifically the contribution of Dt) on inflammation prior to loading the hydrophobic drug of interest. Recent studies have shown that hydrogels composed of bioactive natural polymers can inherently modulate inflammatory processes without external drug loading, supporting the relevance of preliminary drug‐free evaluations [49, 50, 51]. Further studies, including histopathological evaluations, are planned to provide mechanistic insights and confirm edema reduction at the tissue level. A digital caliper was used to measure the thickness before and 2 h after inflammation was induced. The difference in thickness before and after xylene application was calculated. The following formula was used to determine the percentage of inhibition of ear edema:

(5) $$Inibitionpercentage\left(\%\right)=\frac{\left(D_{n}-D\right)}{D_{n}}\times 100$$

*D* refers to the difference in ear edema thickness in the treated group, and *D*_n_ refers to the difference in ear edema thickness in the negative group.

### 2.8 Drug Loading

To achieve equilibrium swelling, pre‐weighed HFs (∼200 mg) were immersed in 50 mL of an 8:2 methanol/water mixture containing ECN (5 mg/mL) [10, 40]. This concentration was chosen to balance the necessity for a sufficient concentration gradient to promote diffusion into the hydrogel network with ECN solubility in the methanol/water mixture. The drug‐loaded HF was dried for 24 h at 40°C in a hot air oven. A small piece of drug‐loaded hydrogel was divided into portions, weighed, and submerged in 50 mL of methanol/water (8:2) to determine the amount of ECN loaded. The drug‐loading efficiency (%) was calculated as (amount of drug loaded/initial amount of drug available) × 100. The encapsulation efficiency (%) was defined as (amount of drug retained in the hydrogel / theoretical maximum capacity) × 100. The amount of ECN released in the solution was assessed using spectrophotometry (*λ* = 225 nm) after stirring the mixture for 24 h with a magnetic stirrer. Complete extraction was validated by repeated washing until no further absorbance was detected at 225 nm wavelength.

### 2.9 In Vitro Drug Release Study

Dry HFs loaded with ECN (approximately 50 mg) were submerged at 25°C in 10 mL of Tris‐HCl buffer (pH 7.4) containing 0.5% sodium lauryl sulfate (SLS). SLS was used to maintain the sink condition. Samples were regularly removed and replaced with a new medium to maintain a constant volume of liquid (dissolution medium). The amount of ECN released in the samples was determined using spectrophotometry (*λ* = 225 nm) in triplicate [40]. As a baseline correction blank, a Tris‐HCl buffer containing 0.5% w/v sodium lauryl sulfate (SLS) was used [41].

### 2.10 Statistical Analysis

All determinations were conducted in triplicate, and the data were subjected to one‐way analysis of variance (ANOVA) followed by Tukey's multiple comparison tests. Results are expressed as the mean ± standard deviation (SD), and half‐maximal effective concentration (EC_50_) values are given as geometric means with 95% confidence intervals (CI). Statistical analysis was performed using Student's *t*‐test for significance with GraphPad Prism‐6 (GraphPad Software, San Diego, California, USA, http://www.graphpad.com). Differences were considered statistically significant at *p* ≤ 0.05.

## 3 Results and Discussion

### 3.1 Formation of CMC‐Dt HFs

The pre‐formulation batches were prepared to maximize the CA content and the cure temperature and time for the HF. To eliminate unreacted polymer and CA, HF was cleaned with distilled water and isopropyl alcohol. The Dt to CMC ratio was adjusted between 1:1 and 2:1, while maintaining the total polymer concentration at 2%. Previous research on the preparation of CMC hydrogels cross‐linked with polycarboxylic acids reported curing temperatures of 80°C and curing times ranging from 3 to 24 h [28, 33]. Other studies systematically compared curing at higher temperatures (e.g., 160°C) for different durations (20, 30, and 40 min) and highlighted the effects of these conditions on hydrogel properties [1].

Based on previous research indicating optimal cross‐linking efficiency and thermal stability for similar polysaccharide systems [31], a curing condition of 140°C for 5 min was selected. This temperature and duration ensured effective esterification while avoiding degradation of the Dt and β‐CD components, as confirmed by TGA, which showed stability up to 200°C. Although minor sugar caramelization cannot be fully excluded, no significant degradation was detected under these conditions. However, 20% CA (polymer concentration) was required for effective cross‐linking of the hydrogels.

CA and polysaccharides (CMC and Dt) formed cross‐links owing to the esterification reaction. Dt acts as both a chemical and physical contributor. Chemically, the hydroxyl‐rich polysaccharide component of Dt can participate in esterification with CA, forming additional intermolecular bonds within the polymeric network. The fibrous matrix of Dt contributes to the hydrogel's structure, potentially influencing its swelling capacity and mechanical integrity, thereby facilitating drug loading and release. Cyclic anhydride intermediate, formed when CA is heated, serves as the fundamental process by which cross‐links with Dt and CMC are formed. In addition to CMC‐Dt cross‐links, the hydrogel matrix may also contain Dt–Dt and CMC–CMC cross‐links. The cyclic anhydride intermediate produces new carboxylic acid units in CA by esterifying the–OH functional groups of the polysaccharide. These units can combine with adjacent carboxylic acid units to form an intramolecular anhydride [28, 39]. As the primary‐OH groups of polysaccharides are more reactive than secondary‐OH groups during esterification, they may also be involved in this reaction [52]. Cross‐linking usually requires high temperatures and a dry environment [42]. However, water evaporation, the first stage of cross‐linking with CA, is facilitated by the substrate's hydrophobicity [52]. The thickness of the developed HFs ranged from 180 to 190 µm. Variations in the polysaccharide ratio may affect the thickness of hydrogel dressings.

### 3.2 Active β‐CD Content

The β‐CD content was found to depend on the β‐CD concentration in the feed. The molecules with non‐sterically hindered cavities are active β‐CD molecules. Phenolphthalein can form an inclusion complex with β‐CD, and its dianion is colorless in alkaline solution [53]. Only the active β‐CD molecules in the HFs can form inclusion complexes with hydrophobic drugs. Active β‐CDs are crucial for enhancing loading and regulating the release of hydrophobic medications from the hydrogel matrix. This technique is only helpful for determining the concentration of active β‐CD molecules that provide guest molecules with accessibility to their cavities. CA facilitates esterification between the primary hydroxyl groups of β‑CD and the C6‑OH groups of CMC/Dt, potentially leading to covalent graft formation. Repeated washing with water and isopropanol removes the physically entrapped β‑CD. The phenolphthalein assay indicated the presence of accessible β‐CD cavities that facilitate drug inclusion. However, this assay alone does not provide definitive evidence of covalent grafting. While the data are consistent with potential β‐CD incorporation via CA‐mediated esterification, further chemical characterization, such as solid‐state NMR or elemental analysis, is required to quantify grafting efficiency. This limitation will be addressed in future studies to comprehensively elucidate the grafting mechanism and its impact on drug loading and release. Table 3 presents the active β‐CD content of HFs. Research findings indicate that when CMC is used alone, it exhibits poor cross‐linking. This is due to the positions of the hydroxy groups at C6, C2, and C3. Additionally, interpolymer cross‐linking is challenging due to electrostatic repulsion between the COO− groups on neighboring polymer chains. This results in interpolymer cross‐linking, which joins the hydroxy groups of the same polymer chain [33, 54]. Published reports have shown that 20% CA provides sufficient cross‐linking between CMC chains. Moreover, the basic nature of the primary hydroxy groups of β‐CD makes them highly reactive toward esterification. The addition of β‐CD to CMC‐Dt HFs allows the primary hydroxy groups of β‐CD to link to the C6‐OH groups of CMC‐Dt, forming a graft‐type I [41]. Therefore, a greater percentage of β‐CD was hypothesized to increase the active β‐CD content and may potentially improve β‐CD‐CMC cross‐linking. Accordingly, our discussion refers to β‑CD incorporation, noting that accessibility, rather than covalent substitution, is directly measured by this method.

**TABLE 3:** TCC, active β‐CD content, and drug loading of HFs.

Batches	TCC (mEq/100 g of hydrogel)	Active β‐CD content (mg/g of HF)^*^	Drug loading^*^ (mg/g)
F1	310	7.16 ± 2.23	25.92 ± 0.69
F2	270	/	25.19 ± 0.26
F3	330	/	25.80 ± 1.05
F4	350	5.26 ± 2.81	28.85 ± 1.53
F5	190	/	25.86 ± 1.16
F6	270	12.29 ± 3.07	25.36 ± 2.01

^*^Mean ± standard deviation.

### 3.3 Total Carboxyl Content (TCC)

Table 3 displays the HF's carboxyl content. In this regard, the numbers indicate the number of esterified and free COOH groups in CA [40]. TCC values can be used to evaluate the rate of cross‐linking in CMC‐Dt HFs [41]. Our findings showed that the average carboxyl content in F1 and F2 was higher than in other films. In addition, an increase in Dt in the presence of β‐CD was accompanied by an increase in the carboxyl content of the HFs. Films in F4 exhibited the highest carboxyl concentration at 350 mEq/100 g hydrogel, which may be attributed to a preference for intercross‐links over intra‐cross‐links in Dt, or to Dt's elevated polysaccharide content. This could also be attributed to Dt's increased involvement in the CA esterification reaction [10].

In contrast, F5 showed a minimum carboxyl content of 190 mEq/100 g hydrogel compared to the CMC‐Dt HFs; the F5 HF has fewer interpolymer cross‐links. The degree of substitution (DS) of CMC and the minimal electrostatic repulsion between adjacent CMC chains, resulting from the F5 feed with few COO‐ groups, can explain this phenomenon. According to the DS, some primary hydroxyl groups (C6‐OH) on the CMC are available for esterification. However, CA may be prevented from joining nearby CMC chains by weak electrostatic repulsion. Consequently, most CA remain unused [38]. Our results indicated that the decrease in TCC in F3 and the increase in TCC in F6 may be due to the simultaneous absence and presence of β‐CD, respectively. Furthermore, our results showed that carboxyl content decreases with increasing β‐CD concentration. This could be explained by the formation of β‐CD‐CA‐βCD conjugates [40], where the β‐CD increases the TCC. This could be due to the formation of (Dt‐CA‐β CD/CMC‐CA‐Dt/Dt‐CA‐Dt) or cross‐links between polymer and β‐CD. Consequently, the rise in TCC observed in HFs with β‐CD can be explained by CA's nearly equivalent affinity for both the β‐CD primary OH groups and the CMC C6‐OH groups. Published reports showed that CA undergoes an esterification reaction with the C6‐OH groups of CMCs and the major OH groups of β‐CD [28, 41].

### 3.4 Characterization of the HFs

**FIGURE 1:**
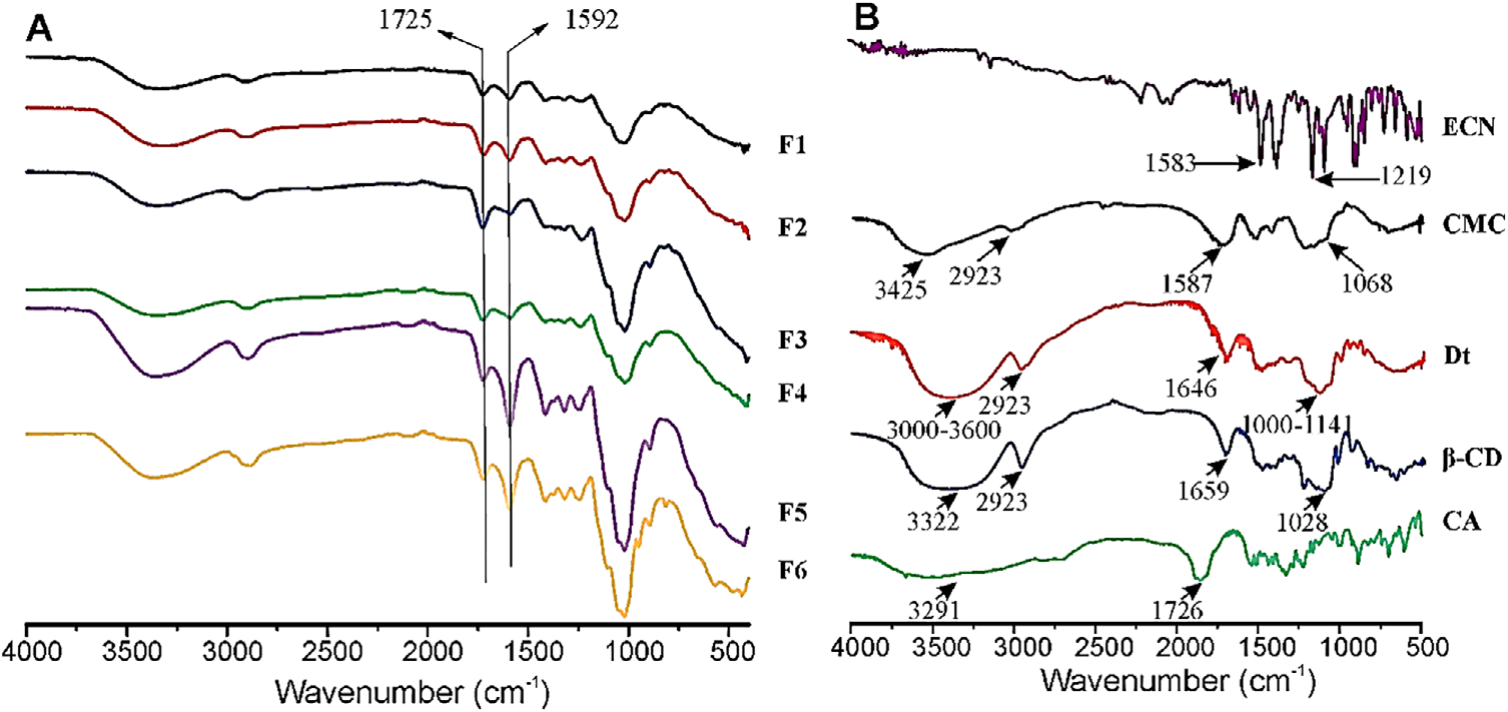
ATR‐FTIR spectra of HFs (A) and ATR‐FTIR spectra of carboxymethyl cellulose (CMC), Dates (Dt), CA, β‐CD, and ECN (B).

FT‐IR spectroscopy is an easy‐to‐use method for quickly obtaining information on the composition, chemistry, and interactions of composite materials [55]. This technique was used to characterize HFs. The ATR‐FTIR spectra of the HFs (F1–F6) are shown in Figure 1A, and the spectra of CMC, Dt, the pure drug (ECN), β‐CD, and CA are shown in Figure 1B. The ATR‐FTIR spectrum of Dt showed large prominent absorption bands at 3000–3600 cm^−1^ attributed to the stretching vibration of the hydroxyl group ─OH. In addition, the spectrum shows strong bands at 1000 and 1141 cm^−1^, possibly due to the superposition of carbohydrate C─O and C─H vibrations. The band that appeared at 2923 cm^−1^ is due to the asymmetric CH stretching, whereas the peak at 1646 cm^−1^ is caused by the stretching vibration of the carbonyl (─C═O) or might also be associated with the H─O─H deformation vibration of the bound water. In contrast, a prominent peak at 3291 cm^−1^, attributed to ─OH stretching, and a sharp peak at 1726 cm^−1^ due to hydrogen‐linked C═O stretching, were observed in the CA spectra.

The spectrum of CMC showed characteristic peaks at 3425 cm^−1^ (─OH stretching), 2923 cm^−1^ due to C─H stretching vibration, and sharp peaks at 1587 and 1408 cm^−1^ caused by the COO^−^ group's symmetric and asymmetric stretching. In addition, the spectrum showed absorption bands at 1068 cm^−1^ (C─O─C stretching) and 1323 cm^−1^ (CH─O CH_2_ stretching) [33]. The β‐CD spectrum exhibits strong absorption bands at 3322 cm^−1^ (O─H stretching), 2923 cm^−1^ (C─H stretching), 1659 cm^−1^ (H─O─H bending), and 1028 cm^−1^ (C─O─C stretching). In contrast, the spectrum of ECN showed characteristic peaks of ECN at 2963 cm^−1^ (C─H stretching), C─C stretch at 1481 cm^−1^, C─H stretching for aromatic at 3108 cm^−1^, C═C stretching for aromatic at 1583 cm^−1^, ─NO_2_ stretching at 1547 cm^−1^, C─O stretching for ether at 1090 cm^−1^, C─O─C stretching at 1219 cm^−1^, and C─Cl stretching at 638 cm^−1^ [56, 57].

**FIGURE 2:**
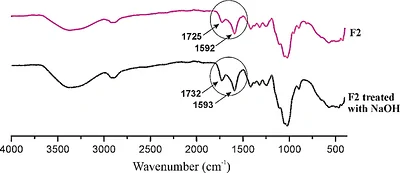
ATR‐FTIR spectra of batches before and after NaOH treatment.

The ATR‐FTIR spectra of the hydrogels showed additional peaks between 1715 and 1730 cm^−1^, attributed to carbonyl bands of free carboxylic acid groups and ester carbonyl groups, suggesting possible cross‐linking interactions within the hydrogel network. The band at 1592 cm^−^^1^ corresponds to the C═O stretching mode of the CMC carboxylate group. Although the ATR‐FTIR spectra of the HFs show evidence of ester bond formation, they are not sufficient to verify the covalent grafting of β‐CD. Solid‐state NMR, XRD, and elemental analyses will be used in future research to determine the degree of substitution and grafting efficiency. The HF was treated with 0.1 N methanolic NaOH for 1 h to separate the ester carbonyl from the acid carbonyl peak, supporting the presence of ester bonds. In the presence of NaOH, free carboxylic acid groups (COOH) are converted into carboxylate anions, which exhibit absorption at lower wavenumbers [42]. The spectra of F2 before and after NaOH treatment are displayed in Figure 2. The spectrum shows that the carbonyl band at 1725 cm^−^^1^ disappears from the spectrum of F2, which was treated with NaOH. A weak peak corresponding to the ester carbonyl band appeared at 1732 cm^−1^, whereas the carbonyl group of carboxylates displayed an absorbance at 1593 cm^−1^, confirming cross‐linking. Their cross‐linking density decreased, as evidenced by the reduced peak intensity at 1725 cm^−1^.

**FIGURE 3:**
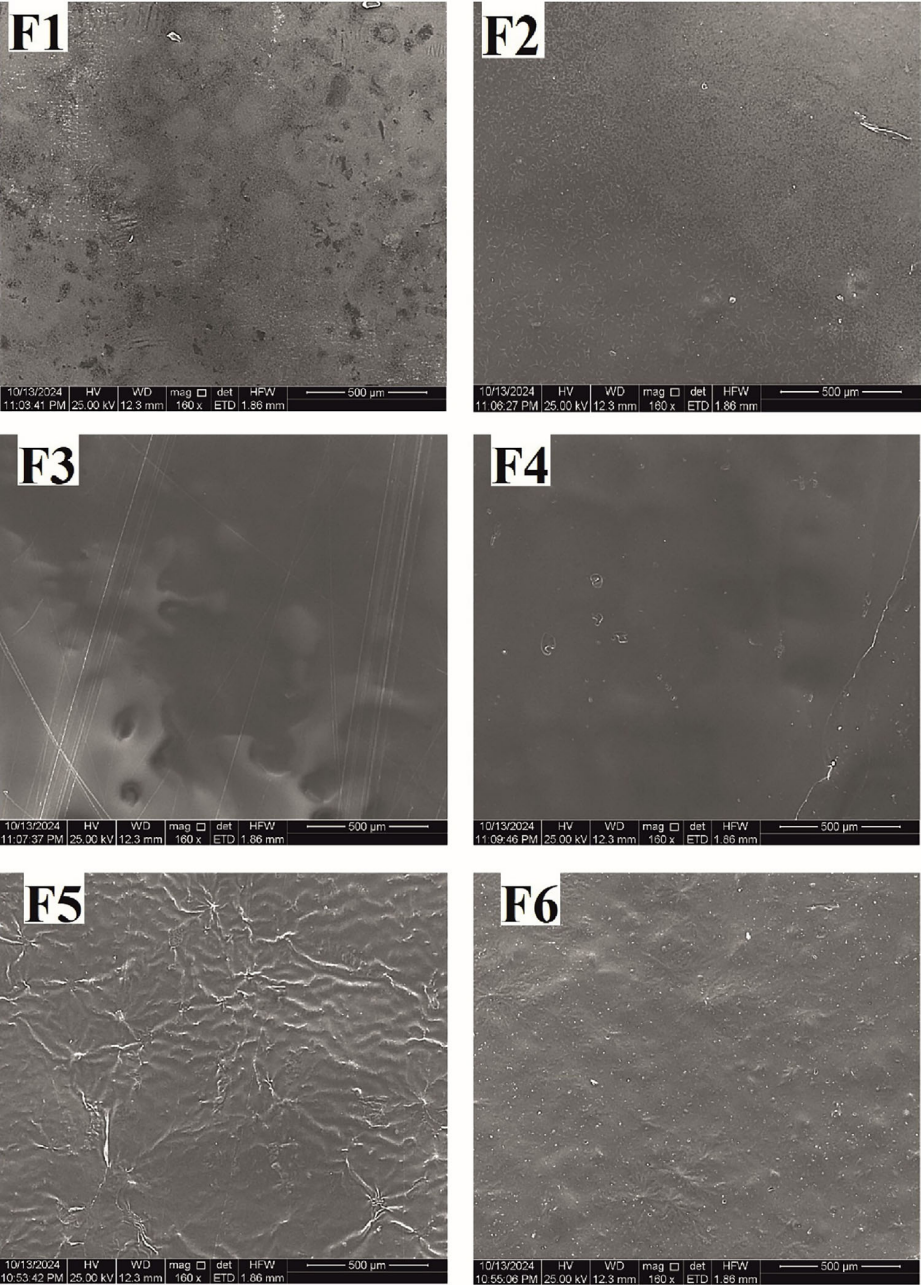
SEM photomicrographs of HFs.

The surface morphology of the HF was investigated using a scanning electron microscope (SEM), as shown in Figure 3. The SEM images revealed a rough surface with pores and/or fissures. However, the SEM of HFs containing β‐CD shows fewer wrinkles, and smooth particles exhibit a white texture, possibly due to reduced cross‐linking density and β‐CD incorporation, compared with photomicrographs of HFs without β‐CD. Results also demonstrated that a smooth surface plays a significant part in the stability of the polymer network. Wrinkles were observed in the HFs without β‐CD, which may be explained by the high cross‐linking density. Pore structure was a key factor in water diffusion and drug release [58]. The two types of surfaces have different roles but are complementary in their polymer properties. Moreover, the HF's roughness and porosity decreased with increasing Dt concentration in F3 and F4 compared with F1 and F2. This might result from increased film cross‐linking density, which suggests that the cross‐linking reaction involves Dt. Consequently, the density of cross‐linking, β‐CD content, and Dt concentration determined the surface morphology of HF. The SEM images show that the HF's surface is covered with small pores.

**FIGURE 4:**
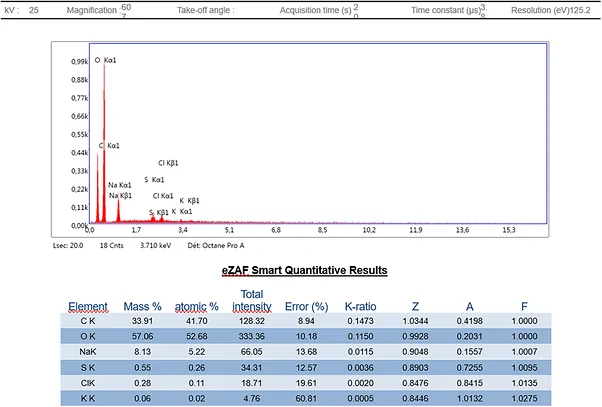
Elemental composition of the HF determined by EDX analysis.

The elemental composition of the HFs was analyzed using EDX, as shown in Figure 4. According to the EDX analysis, the predominant elements of the HFs were carbon (C) and oxygen (O), consistent with the organic nature of the composite system (Dt, CMC, CA, and β‐CD). Minor elements such as Na, Cl, K, and S were also observed, likely reflecting the natural composition and variability of Dt and CA. This analysis does not provide a detailed compositional characterization of Dt. Since Dt is a natural material, whose detailed chemical composition has not been fully characterized, future studies, including NMR and other chemical analyses, could help better define its composition and improve reproducibility.

**FIGURE 5:**
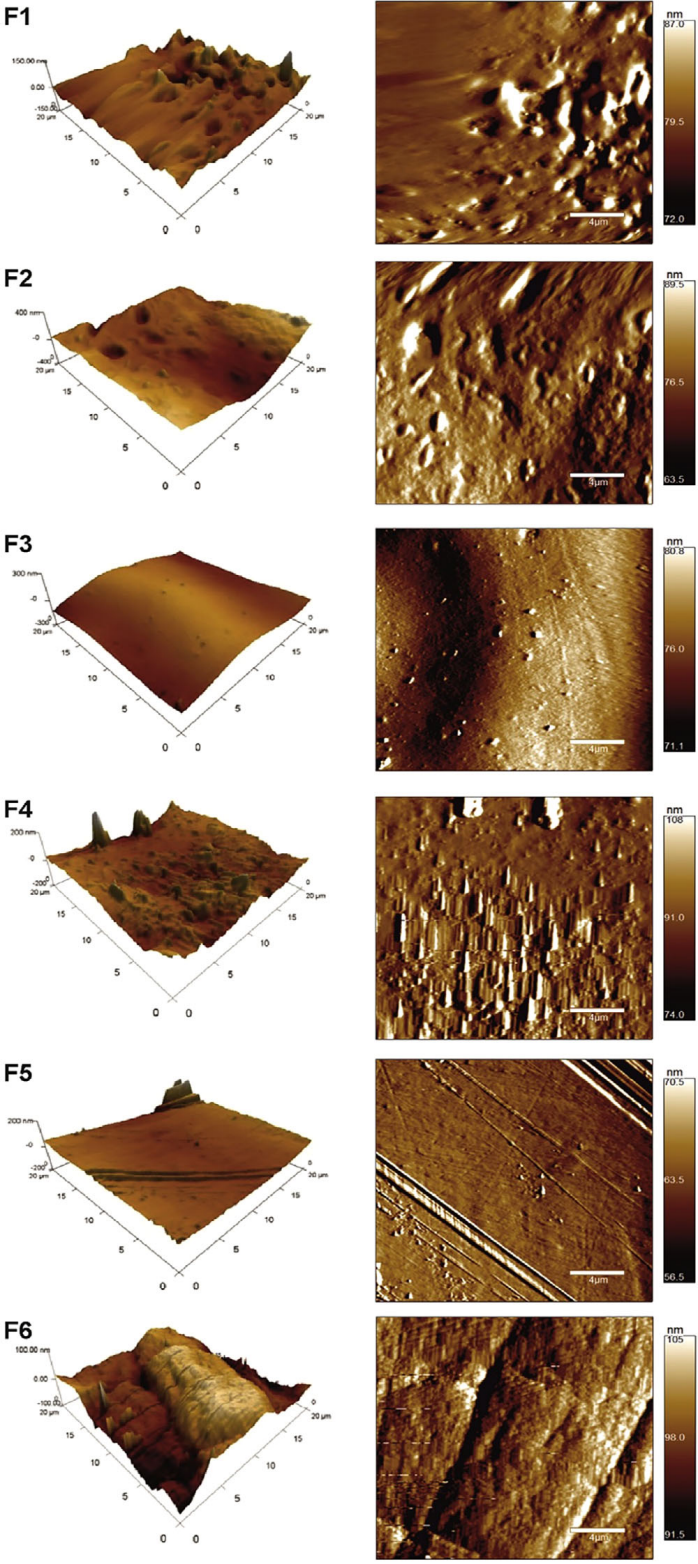
AFM images of cellulose hydrogels F1, F2, F3, F4, F5, and F6.

Visualizing the surface's features was feasible with AFM. The most commonly used roughness measurement in engineering and dentistry is Ra. However, the Ra value does not provide surface profile information; it only gives the average roughness height. On the other hand, RMS (root mean square) is more susceptible to sporadic high and low points. The average surface roughness (Sa) of polymer films determined by the AFM results of polymer samples was 15.37, 62.36, 46.41, 21.07, 18.49, and 27.92 nm for F1, F2, F3, F4, F5, and F6, respectively. Pereira and colleagues reported results comparable to those with cellulose‐based polymer wound dressings, noting a surface roughness of 30.88 nm [59]. In contrast, the RMS values of the films were 21.49, 82.07, 52.01, 27.58, 24.49, and 33.12 nm for F1, F2, F3, F4, F5, and F6, respectively. Figure 5. Depicts the AFM images of cellulose hydrogels F1, F2, F3, F4, F5, and F6. The phase micrographs demonstrate the hardness of the prepared materials and validate the rough surface for all samples. The HF's rough surface may aid adhesion to healing tissue and promote cellular activity, thereby accelerating wound healing [60].

**FIGURE 6:**
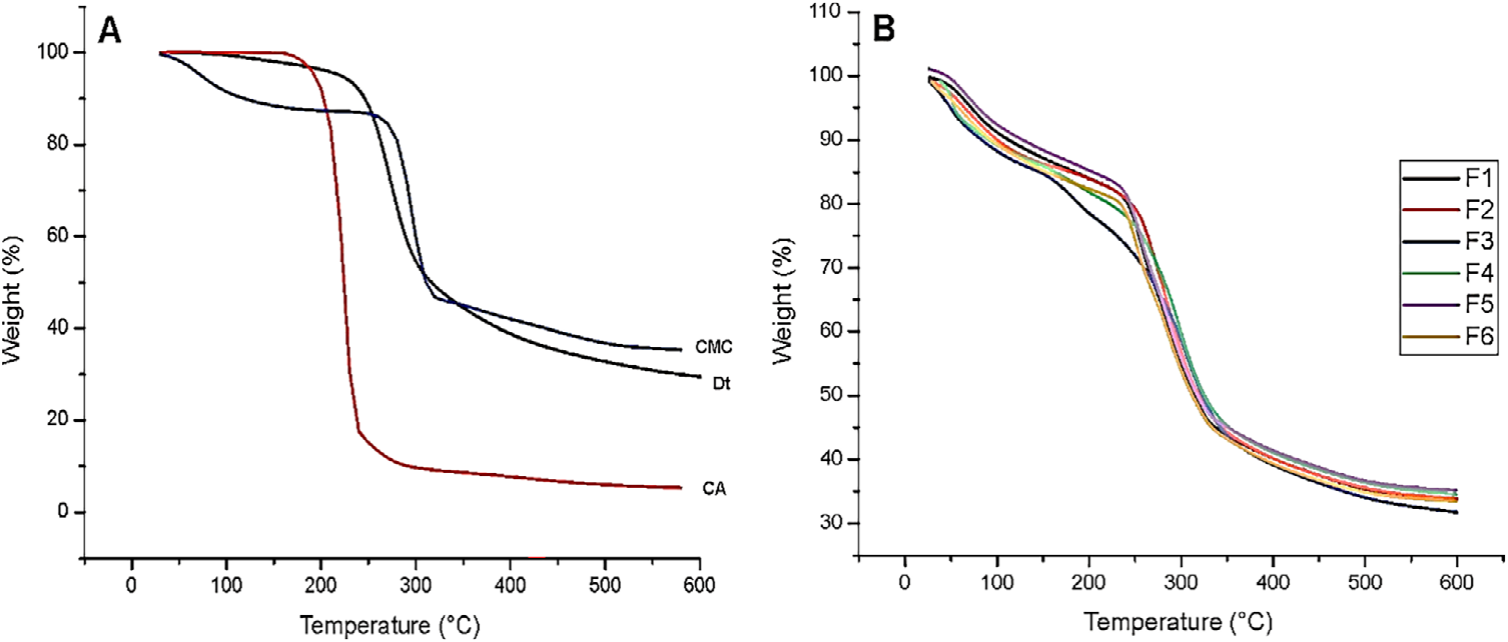
TGA thermograms of CMC, CA, and Dt (a), TGA thermograms of the HFs (F1–F6) (b).

TGA was performed on samples and CMC, Dt, and CA. Figure 6A depicts the thermograms of CMC, Dt, and CA, whereas the thermograms of F1, F2, F3, F4, F5, and F6 are displayed in Figure 6B. The thermograms revealed two stages of decomposition, as observed in the CMC TGA. The first stage, which starts at 30°C and ends at 90°C, has a mass loss of 7.24% attributed to the loss of the polymer's free and bound water. In contrast, the second stage is characterized by a 40.99% mass loss between 227°C and 328°C, due to thermal degradation of the sample. Our findings also showed that the decomposition of anhydrous CA occurs between 135°C and 335°C, with a 91% mass loss. For Dt, there are two stages of decomposition seen in the Dt thermal decomposition curve, which starts at 40°C and finishes at 145°C, with a mass loss of 1.97% ascribed to the removal of bound and free water from Dt; this was followed by a 56.7% mass loss between 228°C and 380°C. At 595°C, the overall weight loss was 71%. Dt's organic components' breakdown and degradation are responsible for the second stage's weight loss.

The TGA thermograms of the HFs show two degradation regions up to 600°C. The first region, between 35 and 175°C, with a 14% weight loss, corresponds to moisture loss and water removal from the hydrogels. This water may be trapped in the β‐CD cavity or related to the hydrogels' network structure. The HFs containing β‐CD exhibited significant weight loss, indicating their hydrophilicity and suggesting the formation of a complex 3D network between the β‐CD and CMC chains [6]. Furthermore, the CA present in the HFs may form cyclic anhydrides as the temperature increased during the analysis. This may be due to the elimination of hydroxyl groups (─OH) and the liberation of water [59]. The second stage of weight loss was observed at approximately 200°C–400°C with a 42% weight loss, which is caused by the decomposition of the glucose units of cellulose and potentially β‐CD as well; this is similar to that reported by other researchers [61] and may be due to the decomposition of the CA cross‐linked CMC‐Dt HFs. At 473°C, the weight loss was 76%. The TGA thermograms show the improved thermal stability of the hydrogel formulations. This behavior may be attributed to enhanced interactions within the polymeric network, possibly indicating an increase in the cross‐link density. Similar findings have been reported in previous studies, where enhanced thermal stability was associated with increased cross‐linking in polymer‐based systems [62, 63]. However, this interpretation is based on indirect evidence from TGA analysis. Further investigations, particularly using NMR, will be conducted to confirm the proposed mechanism.

### 3.5 Determination of Swelling Ratio

**FIGURE 7:**
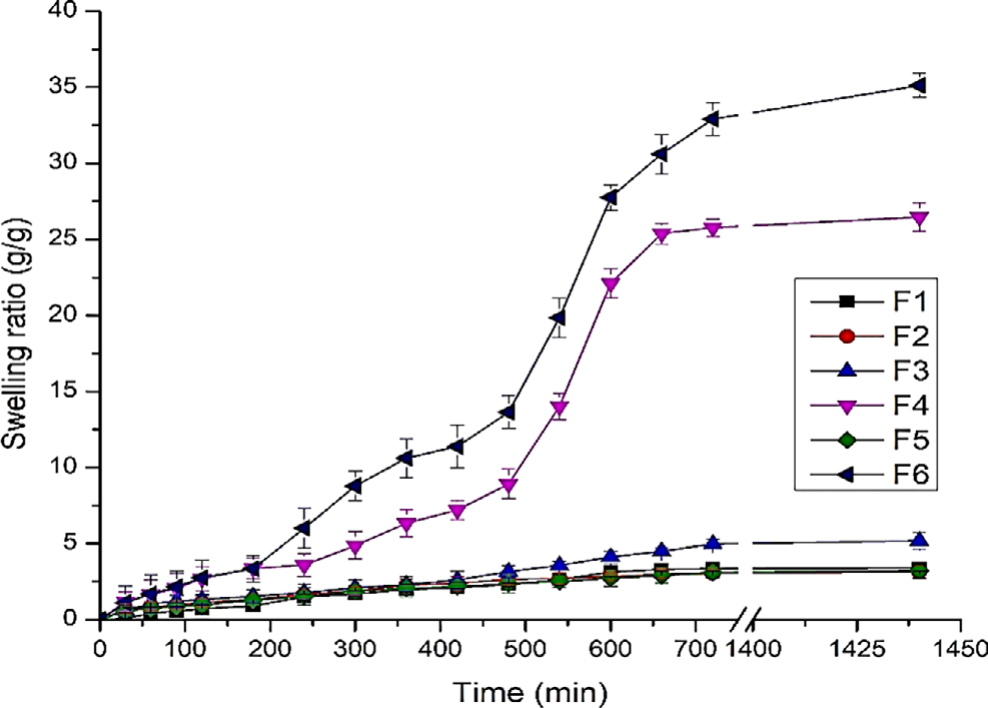
HF swelling profile (F1–F6).

For this, we used a 0.1N Tris‐HCl solution (pH 7.4), and the results are displayed in Figure 7. The results indicated that the SR increased initially and reached equilibrium in all films, and the degree of cross‐linking and concentration of beta‐CD influenced HF's swelling capacity. Owing to air retention within the HF, all films exhibited a markedly reduced initial swelling rate. The results also showed that F6 exhibited a maximum equilibrium swelling of 30.66 ± 2.30 g/g, whereas F2 and F5 displayed the lowest amounts of equilibrium swelling of 2.98 ± 0.95 g/g and 2.89 ± 0.51 g/g, respectively. Cross‐links prevent the hydrogel network from expanding upon contact with the medium, leading to an elastic response that affects HF swelling [64]. After 10 h, all hydrogel batches displayed a constant SR. The β‐CD content, degree of cross‐linking, and ratio of polymer to Dt were related to the SR of the HFs at equilibrium. Results also revealed that when the CMC‐Dt ratio was equal (F1 and F2), and a close equilibrium SR of 3.31 ± 0.90 g/g and 2.98 ± 0.95 g/g was simultaneously achieved.

The difference in swelling may be due to the absence of β‐CD in F2. Because β‐CD is hydrophilic, the hydration of the HF increased with its concentration. Furthermore, as the β‐CD content increased, the interpolymer cross‐linking density decreased, allowing many water molecules to be incorporated into the HFs [40]. In contrast, our findings showed that as the Dt ratio was increased, without β‐CD, the equilibrium SR was found to be lower in the case of F3; this may be due to the absence of β‐CD and the increase in interpolymer cross‐linking density, causing a decrease in the network space and mobility of polymer chains, thus causing a reduction in the SR. When the Dt ratio was increased with β‐CD, there was an increase in the equilibrium SR in the case of F4. This could be explained by the HF's high Dt and low CMC content, which reduced the ionic character and increased HF's hydrophilicity [10]. Furthermore, F4's swelling ability can be enhanced by increasing the carboxyl content. The carboxyl groups may strengthen the hydration and hydrogen‐bonding interactions between HF and water, thereby enhancing the HFs' capacity to swell.

F6 (Figure 7) exhibited the maximum equilibrium swelling. This increase in the SR could be explained by a decrease in interpolymer cross‐linking and an increase in β‐CD content, or due to CMC's high hydrophilicity, which increases water absorption, thus increasing the SR [65]. An increase in the concentration of free and esterified carboxyl groups within the hydrogels is associated with an increase in TCC, which could be another factor contributing to the SR increase in F6. Free carboxyl groups ionize to generate carboxylate ions (COO^−^) when they encounter water, thus increasing the SR because of the electrical repulsion between the ionized acid groups (–COO^−^), which produces a greater swelling force [66]. In contrast, the significant decrease in the SR of F5 may be due to the absence of β‐CD and a decrease in the amount of carboxyl because CA thermally breaks down.

### 3.6 Xylene‐Induced Ear Edema

**FIGURE 8:**
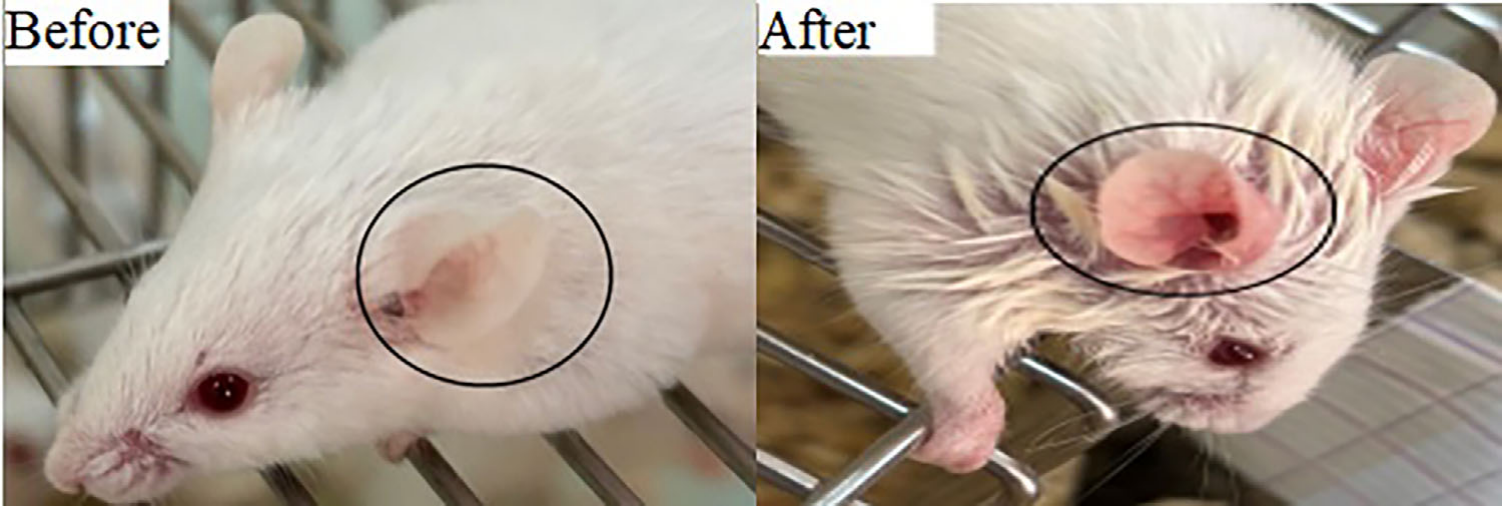
The ear thickness of the mice was measured before and 2 h after the local administration of xylene.

**FIGURE 9:**
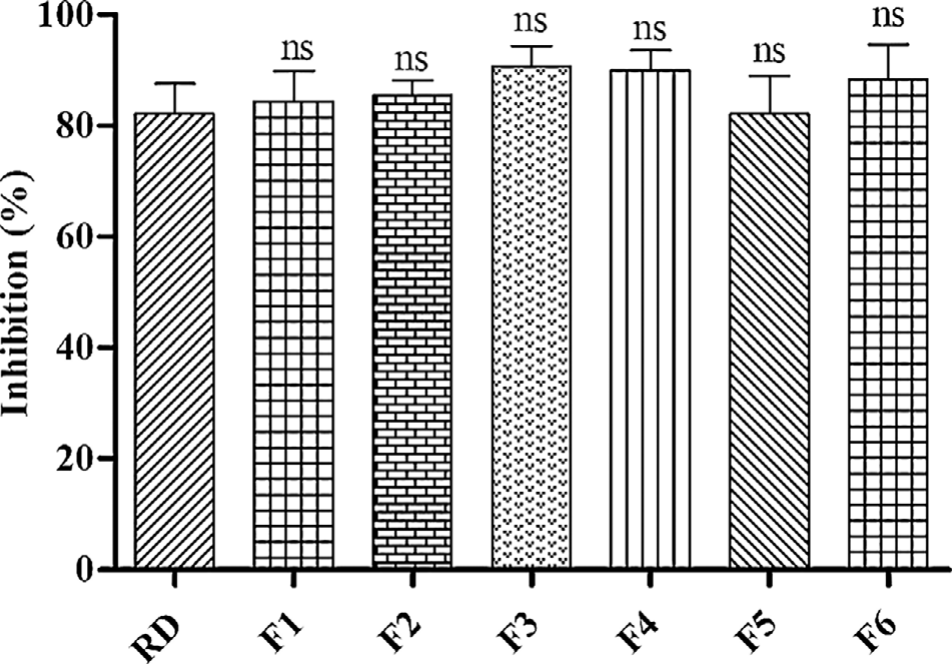
Percentage inhibition of ear edema in mice after 2 h of hydrogel treatment. Results are shown as mean ± SEM (*n* = 6). ns: no significant difference. Statistical analysis was conducted using a one‐way analysis of variance (ANOVA) test.

Mice with xylene‐induced ear edema served as a model of acute inflammation and were used to assess the anti‐edematous effects of the hydrogel samples. Xylene‐induced ear edema provides a consistent, high‐predictive‐value experimental model for screening anti‐inflammatory drugs. The application of xylene to the ear causes fluid accumulation, resulting in edema, a sign of acute inflammation [67]. In the present investigation, the anti‐edema impact of the samples under investigation was determined by comparing the ear thickness of the mice before and 2 h after the local administration of xylene (Figure 8). Our findings showed that all hydrogel samples were able to reduce inflammation in xylene‐induced ear edema to a certain degree, as shown in Figure 9. The effects of F1, F2, and F5 were comparable to those of the standard anti‐inflammatory drug. Our findings indicate that F3 and F4 at high Dt concentrations reduced the edematous response by 92% ± 5.07% and 89% ± 3.69%, respectively. However, the inhibition percentage in the anti‐inflammatory drug model was 82% ± 2.75%. Dt contains phenolic compounds, flavonoids, and other antioxidants. By inhibiting pro‐inflammatory mediators and eliminating reactive oxygen compounds, these molecules reduce inflammation and contribute to the observed decrease in edema. In our study, the presence of β‐CD showed no effect on reducing inflammation in F1, F4, and F6 compared to samples containing β‐CD in F2, F3, and F5.

The anti‐inflammatory properties of the formulated CMC‐Dt HFs can be attributed to the combined action of the hydrogel matrix and the ECN delivery system, which helps maintain therapeutic drug levels at the site of application without the need for frequent drug administration [68, 69]. The hydrated CMC‐Dt hydrogel matrix creates a moist environment for tissue healing, which also helps reduce local irritation and control inflammation during the wound‐healing process [70, 71]. The swelling properties and porosity of the hydrogel matrix enable controlled drug delivery of ECN [68, 69]. In addition, hydrogels as wound dressings have been shown to control excessive inflammation by regulating immune cell activity, reducing oxidative stress, and promoting tissue regeneration, thereby facilitating a rapid shift from the inflammatory to the proliferative phase of the wound‐healing process [68, 70]. Despite the encouraging anti‐inflammatory effects of the current study, the exact molecular mechanism was not elucidated. Thus, further research is necessary to assess levels of inflammatory biomarkers, such as TNF‐α, IL‐1β, IL‐6, COX‐2, and the NF‐κB signaling pathway [69, 71]. Hydrogel dressings have been extensively studied for their high water content, biocompatibility, cooling properties, and ability to create a wet wound bed that facilitates cell migration and tissue regeneration. Commercial hydrogel dressings offer an advantage in decreasing pain and wound‐bed hydration. Nevertheless, traditional hydrogels are characterized by low mechanical strength, poor deformability, limited adhesion to uneven surfaces, and low exudate‐absorptive capacity. Thus, recent investigations focus on multifunctional hydrogels that exhibit enhanced mechanical strength, antimicrobial, antioxidative, self‐healing, and sustained‐release properties [70].

### 3.7 Drug Loading and Release

At pH 7.4, in vitro drug release was performed to assess the potential of the synthesized HFs for the topical treatment of burns and wounds or as implants. Table 3 presents the in vitro drug release profiles of ECN from the HFs. Results show that drug loading affects the number of carboxyl groups, the amount of active β‐CD, and the equilibrium SR (swelling ability) of the HFs. Results also revealed that ECN loading increases with the HFs' swelling ability. More drug molecules can be trapped within the polymer network, enhancing drug diffusion into the hydrogel matrix. In addition to swelling ability, our findings indicated that drug loading decreases in HFs without β‐CD (F2, F3, and F5), which can be explained by the reduced swelling capacity and the absence of β‐CD in the films. There is no formation of an inclusion complex with the drug, which limits the amount of drug that can be loaded; the HFs rely on physical and chemical interactions to retain the drug. Among all HFs, F4 showed the maximum drug loading of 28.85 ± 1.53; for F1 and F6, increasing β‐CD content led to higher drug loading for F1 and greater swelling ability for F6. To prevent the ECN from diffusing out of the hydrogel matrix, the active β‐CD in the HFs may bind to the ECN, forming an inclusion complex.

**FIGURE 10:**
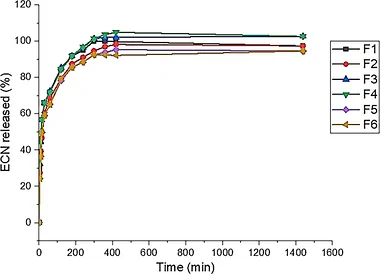
ECN's release profile from the HFs.

Figure 10 depicts the drug release profile of ECN‐loaded HFs. All HFs showed increased ECN release. The initial increase may be due to free drug on the HFs' surface, which is released immediately upon contact with the dissolving liquid. The drug‐loaded HFs exhibited controlled release after an hour. Overall, the study showed that carboxyl content, swelling ability, and active β‐CD content influence the release of ECN from the HFs. Our results show that within the first hours, ECN was released quickly from all samples because the HFs' free carboxylic acid groups produce a somewhat acidic environment. At pH 7.4, the free carboxylic acid groups liberate H^+^ ions through ionization. A mildly acidic environment, created by hydrogels' high concentration of H+ ions, may ionize free ECN molecules, thereby promoting their release. The concentration of H^+^ ions in the hydrogels decreases over time, thereby reducing acidity and considerably slowing the release rate [40]. The release of F2, F3, and F5 was faster in the absence of β‐CD in the HFs formulations, due to decreased carboxyl content. β‐CD stabilizes and controls the drug's release; its absence results in faster uncontrolled diffusion of the drug. According to the literature, the diffusion of drug molecules is often prolonged, and their release is delayed as the hydrogels' swelling capacity increases [72]. In our study, an inverse relationship is observed between F4 and F6, driven by their high SRs and carboxyl content, which increase porosity and solubility. Excessive swelling expands the hydrogel network, creating larger pores and greater water uptake, thereby enhancing drug dissolution and accelerating release.

Drug release profiles fitted to Higuchi, Korsmeyer–Peppas, and zero/first‑order models are shown in Table 4. The Korsmeyer‐Peppas model was used to calculate the amount of drug released over time; it was applied to up to 60% of the total drug released to determine the release mechanism [73, 74, 75]. Results shown in Table 4 indicate that the HFs from F1 to F6 exhibit a quasi‐Fickian release mechanism (*n* < 0.5), demonstrating a low level of interaction between ECN and the HF constituents even with a significant SR. This can be explained by highly porous polymeric networks with large, interconnected pores that enable rapid drug diffusion. The large‐pore structure reduces diffusion resistance, resulting in a generally Fickian release mechanism. To reduce the porosity of the polymeric structure and improve film uniformity, the drying time can be increased, or a second polymer can be added to the HF formulation.

**TABLE 4:** Kinetic modeling and correlation coefficients (*r* ^2^) for drug release profiles according to Higuchi, Korsmeyer–Peppas, zero‐order, and first‐order models.

Batches	Zero‐order	First‐order	Higuchi	Korsmeyer–Peppas	
	*r* ^2^	*r* ^2^	*r* ^2^	*n*	*r* ^2^
F1	0.670	0.339	0.974	0.456	0.945
F2	0.704	0.342	0.989	0.421	0.994
F3	0.652	0.330	0.975	0.433	0.961
F4	0.640	0.325	0.975	0.413	0.969
F5	0.658	0.336	0.979	0.463	0.959
F6	0.657	0.335	0.977	0.454	0.961

The Higuchi model showed the highest correlation coefficient (*r* ^2^ = 0.974–0.989), indicating that drug diffusion through the hydrogel matrix was the principal mechanism of release. In contrast, the comparatively lower *r* ^2^ values for the zero‐order (0.640–0.704) and first‐order models (0.325–0.342) suggest that the release was neither constant nor primarily concentration‐dependent. The diffusion exponent (*n*) from the Korsmeyer–Peppas model, ranging from 0.413 to 0.463, provided complementary insights into the drug transport behavior. This behavior may be attributed to subtle variations in the cross‐link density, matrix swelling capacity, or drug–polymer interactions. The presence of β‐CD likely enhanced drug encapsulation via inclusion complex formation, thereby modulating the diffusional pathways, while CA cross‐linking provided structural integrity and controlled hydration. Collectively, these findings demonstrate that the developed HFs offer a stable, diffusion‐controlled release platform suitable for the sustained topical delivery of ECN.

## 4 Conclusions

In conclusion, the present study's findings indicate the successful preparation of CMC‐Dt HFs in the presence of β‐CD via cross‐linking with CA. The presence of an active β‐CD in the HFs was established using phenolphthalein test, TGA, SEM, AFM, and ATR‐FTIR studies that indicated the formation of ester linkages between the polymer constituents. In addition, TCC increased with increasing Dt concentration. Moreover, the CMC‐Dt HFs demonstrated a high swelling ability, excellent drug loading, and controlled release of ECN. Results of the in vivo study indicated promising anti‐inflammatory properties of the HFs and thus can be considered a potential drug delivery system. Nonetheless, additional studies, including structural characterization by solid‐state NMR, wound‐healing assessment, hemocompatibility testing, and biocompatibility testing, should be conducted to further validate the efficacy and safety of the proposed HFs. Thus, future research should focus on assessing these activities to precisely characterize the overall efficacy of HFs and optimize their use in the pharmaceutical field.

## Author Contributions

**Oumamar Loubna**: conceptualization, formal analysis, investigation, methodology, resources, validation, software, and writing – original draft. **Soltani El‐Khamsa**: methodology, validation, supervision, and writing – original draft. **Kailas Krishnat Mali**: resources, validation, and supervision. **Ahlem Karbab**: data curation, formal analysis, investigation, methodology, software, and writing – original draft. **Noureddine Charef**: formal analysis, validation, and supervision. **Nancy Sowan**: formal analysis and validation. **Mohammad S. Mubarak**: data curation, formal analysis, validation, and writing – review and editing .

## Funding

Neither public nor commercial funding organizations awarded any specific grants for this research.

## Conflicts of Interest

The authors declare no conflicts of interest.

## Data Availability

The data that support the findings of this study are available from the corresponding author upon reasonable request.
